# In vitro and in vivo antibacterial activity of environmental bacteriophages against *Pseudomonas aeruginosa* strains from cystic fibrosis patients

**DOI:** 10.1007/s00253-015-6492-6

**Published:** 2015-03-12

**Authors:** Tomasz Olszak, Paulina Zarnowiec, Wieslaw Kaca, Katarzyna Danis-Wlodarczyk, Daria Augustyniak, Pavel Drevinek, Anthony de Soyza, Siobhán McClean, Zuzanna Drulis-Kawa

**Affiliations:** 1 Institute of Genetics and Microbiology, University of Wroclaw, Przybyszewskiego 63/77, 51-148 Wroclaw, Poland; 2 The Jan Kochanowski University, Swietokrzyska 15, 25-406 Kielce, Poland; 3 Division of Gene Technology, Catholic University of Leuven, Kasteelpark Arenberg 21, 3001 Leuven, Belgium; 4 Department of Medical Microbiology, University Hospital Motol and 2nd Faculty of Medicine, Charles University, 150 06 Prague, Czech Republic; 5 Institute of Cellular Medicine, Medical School, Newcastle University, Framlington Place, Newcastle upon Tyne, NE2 4HH UK; 6 Centre of Microbial Host Interactions, Institute of Technology Tallaght, Tallaght, Dublin 24, Ireland

**Keywords:** *Myoviridae* bacteriophages, Phage treatment, *Pseudomonas aeruginosa*, Cystic fibrosis, *Galleria mellonella* model

## Abstract

The goal of the study was to determine the relationship between in vitro/in vivo efficacy of environmental *Pseudomonas* phages and certain phenotypical properties of *Pseudomonas aeruginosa* (*PA*) strains. We studied the diversity between particular isolates and determined phage sensitivity in vitro and in vivo in the *Galleria mellonella* insect model. Twenty-eight lytic bacteriophages specific for *PA* were tested against 121 CF *PA* isolates including 29 mucoid *PA* strains. Most strains from cystic fibrosis (CF) patients were lysed by at least three phages (93.6 %), but completely insensitive strains were also present (6.4 %). Two phages PA5oct and KT28 exhibited high rates of lytic potency on 55–68 % of *PA* strains (72–86 % of mucoid isolates). We further explored phage activity against six *PA* strains (CF and non-CF) in vitro, comparing clonal differences in phage susceptibility with bacterial properties such as the ability to form biofilms, mucosity, twitching motility, and biochemical profiles. We observed the relationship between variation in phage susceptibility and Fourier transform infrared spectroscopy (FTIR) analysis in the spectra window of carbohydrates. The protective efficacy of two selected phages against *PA* PAO1 and 0038 infection was confirmed in vivo in *G. mellonella* larvae. Generally, the wax moth model results confirmed the data from in vitro assays, but in massive infection of CF isolates, the application of lytic phages probably led to the release of toxic compound causing an increase in larvae mortality. We assumed that apart of in vitro phage activity testing, a simple and convenient wax moth larvae model should be applied for the evaluation of in vivo effectiveness of particular phage preparations.

## Introduction

*Pseudomonas aeruginosa* (*PA*) is the key pathogen associated with lower respiratory tract illness in patients with cystic fibrosis (CF), infecting up to 80 % of adult patients. Once established, the pathogen is difficult to eradicate (Davies 2002; Parkins et al. 2012). Increased viscosity of mucus associated with the CF-lung causes favorable conditions for persistent infection by opportunistic bacteria. Chronic infection and associated inflammatory responses lead to progressive loss of lung function and respiratory failure (Callaghan and McClean 2012; Costello et al. 2011). Antibiotic therapy is usually effective only in the early stages of infection. During the development of a chronic infection, even long-term antibiotic therapy with high doses does not result in eradication of the pathogen. This is multifactorial (Bradbury et al. 2008; Cheng et al. 1996; Fothergill et al. 2012). Bacterial adaptation is a characteristic of CF-related *PA* strains. Alterations in features such as antibiotic resistance, adhesion, alginate, and mucus production are common and can affect the success of clinical treatment (Silbert et al. 2001). Moreover, the strains of *PA* isolated in the late stages of infection are very heterogeneous and demonstrate microevolution within an individual strain with a characterized genetic profile. This phenotypic variability of strains underlies the frequent failure of antibiotic therapy and also creates a problem for the development of alternative treatments (Bragonzi et al. 2009; Cramer et al. 2011). Multidrug resistance and biofilm formation are additional problems associated with the treatment of *P. aeruginosa* infection, implying that alternative treatment methods are needed for eradication or suppression of *PA*. Lytic bacteriophages offer a potentially exciting alternative approach in treatment for *PA* infections. Nevertheless, phage therapy, especially in the eradication of *PA* clones from CF patients, requires extensive preclinical testing of phage biology and antibacterial activity.

In this study, we were interested in the relationship between in vitro/in vivo efficacy of environmental *Pseudomonas* phages and certain phenotypical properties of selected strains.

Therefore, we first selected two phages with the broadest spectrum and analyzed them against six isolates that were examined for mucoid slime production, twitching motility, biofilm-forming ability and biochemical composition variability as analyzed by Fourier transform infrared spectroscopy (FTIR) techniques. Phage lytic activity was tested both in vitro and in vivo using the *Galleria mellonella* larvae model. The most active phages were characterized by electron microscopy and genome size.

## Materials and methods

### Bacterial strains

Eighteen *PA* strains were used as hosts for phage propagation: PAO1 (ATCC 15692) purchased from the American Type Culture Collection (ATCC); two clinical CF *PA* strains (isolated from a CF patient) from the collection of the Prague CF Centre, Czech Republic; one clinical CF *PA* strain (isolated from the lung infection of a CF patient) from the collection of Newcastle University Medical School, Newcastle upon Tyne, UK; and 14 clinical non-CF *PA* strains (isolated from patients without CF) from the collection of the Institute of Genetics and Microbiology, University of Wroclaw, Poland. A total of 123 *PA* isolates were used for phage lytic potency: namely PAO1; 121 clinical CF *PA* isolates from the collection of the Prague CF Centre; and non-CF0038 clinical *PA* strain from the collection of the Institute of Genetics and Microbiology, University of Wroclaw, Poland.

Bacteria were stored at −70 °C in tryptic soy broth (TSB; Becton Dickinson and Company, Cockeysville, MD) supplemented with 20 % glycerol.

### Phage isolation

Six water samples collected from a natural wastewater treatment plant (irrigated fields) located in Wroclaw, Poland, as phage sources were centrifuged at 15,000 *g* for 15 min, and the supernatants were filtered through a 0.22-μm Millex-GP filter (Merck Millipore, Darmstadt, Germany, SLGP033RS). One milliliter of filtered water sample and 0.5 ml of a bacterial broth culture, grown overnight in TSB, were added to 10 ml of TSB and incubated at 37 °C for 18 h. The suspension was then centrifuged again, treated with chloroform, and filtered through a 0.22-μm Millex-GP filter (Merck KGaA, Darmstadt, Germany). This procedure was repeated three times to eliminate any bactericidal activity by contaminating chemicals. Bacteriophage presence and titer in the filtrate were assessed by the plaques test using the double-agar layer technique (Adams 1959). Phages were propagated from a single plaque. The phage lysate was then subjected to PEG 8000 (Acros Organics, Geel, Belgium) precipitation. The 28 environmental bacteriophages lytic on *PA* strains (27 named KT and one phage PA5oct) were deposited in the phage collection of the Institute of Genetics and Microbiology, University of Wroclaw, Poland.

### Phage lytic potency

Prior to phage sensitivity testing bacteria were subcultured in TSB. Unless stated otherwise, bacteria were grown for 4–6 h. To determine bacterial susceptibility to phage-mediated lysis, bacteria grown on liquid TSB medium at 37 °C were transferred directly onto TSA plates. After drying, a drop of the phage suspension (10^8^ PFU ml^−1^) was put on the bacterial layer and incubated at 37 °C. The plates were checked after 4–6 h and again 18 h later for the presence of bacterial lysis. The phage lytic potency assay was repeated at least three times. Spot testing is a rapid and efficient method for determining the host range in a large collection of bacteria (Adams 1959; Kutter 2009).

### Electron microscopy

The filtered high-titer phage lysate was centrifuged at 25,000 *g* for 60 min, using a Beckman (Palo Alto, CA) J2-21 centrifuge and a JA19.1 fixed-angle rotor. The pellets were washed two times in ammonium acetate (0.1 M, pH 7.0) under the same conditions. Phages were deposited on copper grids with carbon-coated Formvar films (Agar Scientific, Elektron Technology UK Ltd, Stansted, UK) and stained for 10 s with uranyl acetate (2 %, pH 4.5) or phosphotungstate (2 %, pH 7). Excess liquid was blotted off, and phages were examined under a Philips EM 300 electron microscope. Magnification was controlled by means of T4 phage tails (Ackermann 2009).

### Pulsed field gel electrophoresis analysis

Pulsed field gel electrophoresis (PFGE) analysis was performed by previously described method (Drulis-Kawa et al. 2014). Prepared blocks were placed in lysis buffer (50 mM Tris, 50 mM EDTA, 1 % SDS) and digested for 2 h with 100-μg ml^−1^ proteinase K solution at 54 °C. After digestion, plugs were rinsed four times with TE buffer. DNA samples were placed on a 1 % agarose gel using the Bio-Rad (Hercules, CA, USA) CHEF-DR III system (16.5 h, 6 V cm^−1^, 12 °C, switch time 1–50, angle 120°). Low Range PFG Marker (New England Biolabs, Ipswich, MA, USA, N0345S) was used as a size marker.

### Subsurface twitching assay

The subsurface twitching assay was performed as previously described by Semmler et al. (1999) with a slight modification. Ten *P. aeruginosa* colonies taken randomly were tested for twitching motility. Each colony was stab-inoculated through the agar to the underlying Petri dish and incubated at 37 °C for 48 h. The zone of motility at the agar Petri dish interface was measured after 0.01 % crystal violet (CV) staining.

### Biofilm quantification by crystal violet assay on peg lid plates

The biofilm formation by *P. aeruginosa* strains was measured by O’Toole and Kolter standard biomass assays using crystal violet (CV) with modifications (Aaron et al. 2002; O’Toole and Kolter 1998; Waszczuk et al. 2012). For the experiments, overnight cultures of bacterial strains were suspended in a physiological saline and diluted to an optical density of 0.5 McFarland units. The culture was then serially diluted in TSB medium to obtain a final density of 5 × 10^5^ colony-forming units (CFU) ml^−1^. Two hundred microliters of prepared suspension was added into each well of a round-bottomed polystyrene 96-well microtiter peg lid plate. The plates were then incubated for 24 h at 37 °C. After 24 h of incubation the peg lid was transferred to new titer plate and washed two times (nonadhered cells were removed from each peg, and lids were rinsed using 200 μl of physiological saline). The peg lids were submerged into 200 μl of 0.01 % CV solution (Sigma-Aldrich Chemie GmbH, Steinheim, Germany) added to each well. The excess CV was removed, and bound CV was released by adding 200 μl of 96 % ethanol (Sigma-Aldrich Chemie GmbH, Steinheim, Germany). The absorbance was measured at 595 nm using a multilabel microtiter plate reader (UVM 340, AsysHitech, Eugendorf, Austria). The positive control consisted of 100 μl of 0.01 % CV solution, while the negative control was 96 % ethanol. All assays were repeated 24 times per strain.

### Attenuated total reflectance–Fourier transform infrared spectroscopy

FTIR is a scientific technique based on measurement of vibrational energy changes of organic compounds excited by IR radiation. Due to the fact that vibrational energy is quantized, the frequencies of various functional groups can be detected only at fixed wavelengths, specific for every molecular compound. Vibrational spectra typical for biological samples in most cases correspond to mid-IR (4000–400 cm^−1^), and there are five spectral windows defined within that spectrum (Alvarez-Ordóñez et al. 2011). For minimal interference with the test material, in this study, the IR spectrum measurement was conducted by attenuated total reflectance (ATR)-FTIR. The IR spectral range was limited to 4000–900 cm^−1^, and four spectral windows were taken into account. Every window was specific for one group of organic compounds, i.e., carbohydrates (1200–900 cm^−1^), carboxylic groups (1500–1200 cm^−1^), proteins (1700–1500 cm^−1^), and lipid compounds (3000–2800 cm^−1^) (Alvarez-Ordóñez et al. 2011).

#### Experimental procedure

The biochemical profile of bacterial samples was measured by ATR-FTIR spectroscopy (Spotlight 400 FTIR Imaging System, Perkin Elmer, Waltham, MA, USA). Sample preparation procedures for ATR include direct transfer of ten single bacterial colonies to the crystal, separately. The spectra were collected at room temperature over the wave space number range of 4000 to 900 cm^−1^ with a resolution of 4 cm^−1^, and 50 repeats were averaged to improve the signal to noise ratio. The spectra were displayed in terms of absorbance, which was calculated using Perkin Elmer software. A background measurement of the crystal was taken before each sample was applied. The spectrum obtained for each clone was analyzed in definite ranges, allowing to estimate the variability of specific groups of biochemical components. The range 1200–900 cm^−1^ is typical for carbohydrates, range 1500–1200 cm^−1^ corresponds to the carboxyl groups, the range of 1700–1500 cm^−1^ is characteristic of proteins and within the 3000–2800 cm^−1^ the lipid fraction can be analyzed.

#### Pretreatment of spectra

The preprocessing was done as follows: baseline correction in the range of 2404 to 2275 cm^−1^ and smoothing of the spectral region from 2725 to 1556 cm^−1^ were done. The spectra were normalized such that the smallest recorded absorbance was set to 0 and the highest was set to 1 for each spectrum and then the first derivatives (Savitzky and Golay) with a window of 5 were used for chemometrics. The derivation of the spectra to the second order was used to increase the number of discriminant features present in the spectra.

#### Chemometric analysis

Reproducibility and discriminatory power were calculated for the working spectrum (range 4000–900 cm^−1^) and, independently, for the particular sections according to the important regions of infrared spectroscopy. To measure the degree of similarity between ten replicates (ten colonies), we used the differentiation index *D* expressed as *D* = (1 − *r*) × 1000, where *r* indicates the Pearson correlation coefficient (Mouwen et al. 2005). Smaller *D* values indicate more similar colonies (for identical strains, *D* = 0). All statistical computations were carried out using the program R version 2.15.1 (R Core Team 2012) (http://www.r-project.org/).

### *Galleria mellonella* larvae model

The in vivo assay was conducted on a wax moth larvae model (*G. mellonella*, Livefoods Direct, Sheffield, UK). Prior to each experiment the larvae were subjected to a 7-day acclimatization period in the dark at 15 °C. Experiments were performed in triplicate (ten larvae per trial). For survival control, we observed both untouched larvae and larvae injected with 20 μl of sterile saline buffer. Depending on the growth rate of the tested bacterial strains, experiments were conducted up to 96 h. Freshly plated bacterial cultures were inoculated into Luria Broth (LB, Sigma-Aldrich) tubes and incubated at 37 °C overnight under agitation. After 18 h, the bacterial suspension was diluted with physiological saline to OD_600_ = 0.1 and this was used as a starting point for serial dilutions. For the measurement of *P. aeruginosa* virulence, 10 μl of diluted bacterial suspension (serially diluted to 10^−8^ CFU) were used to infect larvae. Both bacteria and bacteriophages were administered to larvae by injection into the ventral side of the last pair of pseudopods. After injection, the larvae were incubated for 72 h at 37 °C. The effects of infection were checked at 8, 24, 48, 72, and 96 h after injection by assessment of survival and macroscopic appearance. For assessment of the antibacterial activity of selected phages, larvae were injected with 10 μl of bacterial suspension and, within 1 h, 10 μl of phage lysate at the titration equal to multiplicity of infection (MOI) 100. Each experiment was carried out for 72 h at 37 °C, and results were read at 8, 24, 48, and 72 h. The results were expressed as the percentage survival rates. The experiments were performed at least three separate occasions. The experiments were controlled by observation of uninfected larvae, sham-infected larvae, larvae receiving phage lysate only, and infected but phage untreated larvae.

### Statistical analysis

The analysis of survival curves was performer by log-rank Mantel-Cox test. *p* Values <0.05 were considered statistically significant. Statistical analysis was performed using GraphPad Prism software (GraphPad Software, Inc., La Jolla, USA).

## Results

### Phage characteristics

**Fig. 1:**
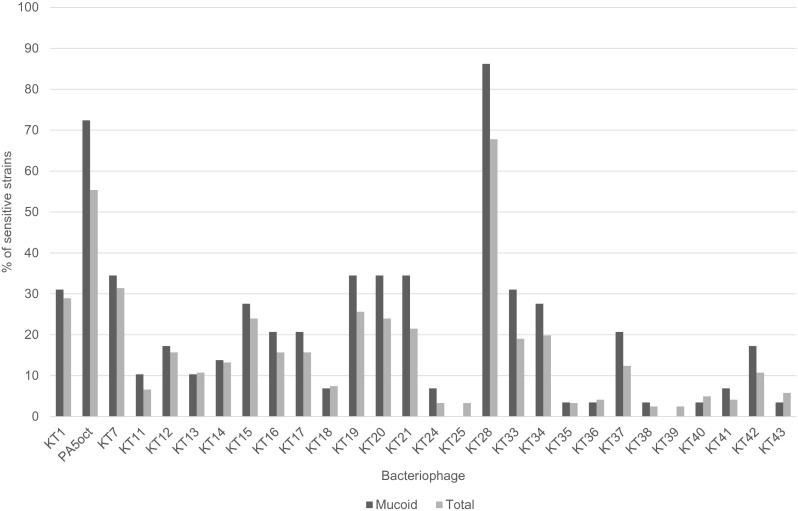
In vitro susceptibility of 123 *P. aeruginosa* strains to the collection of environmental *Pseudomonas* phages. Phage-mediated lysis was performed by spot technique on TSA plates at 37 °C by application of 10^8^-PFU/ml phage suspension. The assay was repeated at least three times

The lytic potency of the phage collection (28) specific for *PA* was performed against 121 CF isolates including 29 mucoid strains. All strains used had been previously characterized by genotyping methods such as multilocus sequence typing (MLST) and random amplification of polymorphic DNA (RAPD). Only unique clones were selected for this study (Maiden et al. 1998). In the phage lytic potency experiments (Fig. 1), several phages exhibited high antibacterial lytic potency, with 55 % (phage PA5oct) and 68 % isolates (phage KT28) in total and 72 and 86 % of mucoid isolates showing potency, respectively. Completely insensitive *PA* were also present (6.4 %). The CF *PA* strains showed strong heterogeneity in phage susceptibility presented as confluent clear lysis, confluent opaque lysis, and single resistant colonies on plaque. Disparate results in three consecutive experiments may suggest the existence of multiple clones within each CF isolate. We have also analyzed isolates, classified to particular RAPD cluster, cultured form one patient at different time of infection. Seventy-one isolates from 31 patients (31 strains total) were typed to evaluate the changes in phage patterns. It turned out that during the colonizing process, 21 strains did not change their susceptibilities to the phage collection, 8 strains lost their ability to propagate phages, and 8 strains become susceptible to more bacteriophages from the collection.

**Fig. 2:**
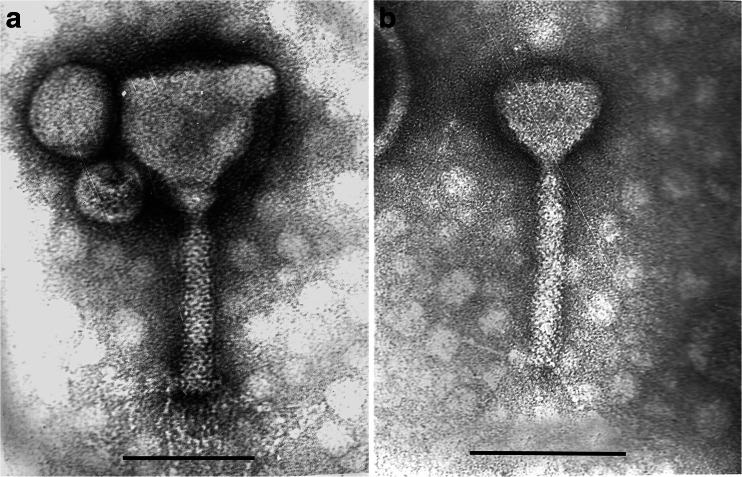
Electron micrograph of PA5oct phage (**a**) and KT28 phage (**b**) negatively stained with uranyl acetate. The *bar* indicates 100 nm

**Fig. 3:**
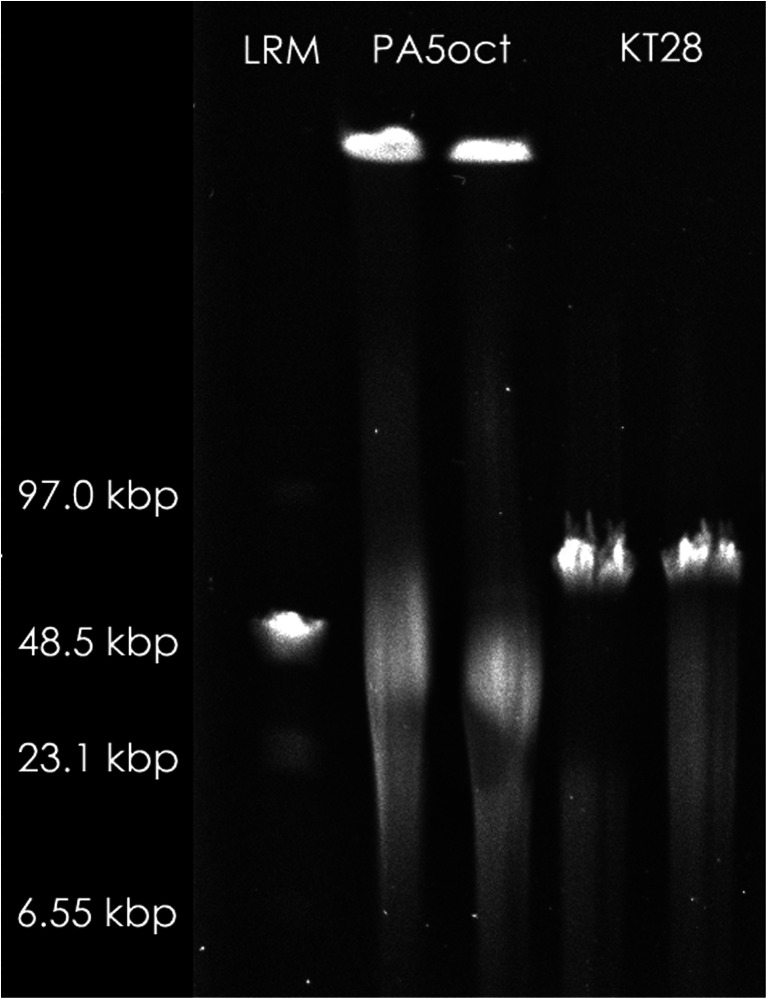
Pulsed field gel electrophoresis analysis of PA5oct phage DNA and KT28 phage DNA. Low Range PFG Marker (LMR) was used as a size marker

The two phages with the strongest lytic activity (PA5oct and KT28) were examined by transmission electron microscopy and classified on the basis of their morphological features in the order *Caudovirales* and the family *Myoviridae* (Fig. 2). Phage KT28 is PB1-like viruses with an approximate head diameter of 70 nm between opposite apices and tails of 113 × 17 nm, a base plate, and fibers of 40 nm in length (Ceyssens et al. 2009). Phage PA5oct is a giant unclassified myovirus with an isometric head of about 131 nm in diameter and a contractile tail about 136 nm long (Drulis-Kawa et al. 2014). The isolates were named according to a recent nomenclature system as vB_PaeM_PA5oct and vB_PaeM_KT28 (Kropinski et al. 2009). The phages’ genome size was determined by PFGE (Fig. 3). KT28 DNAs were about 70 kbp in size, whereas that of PA5oct was approximately 375 kbp (Drulis-Kawa et al. 2014).

**Fig. 4:**
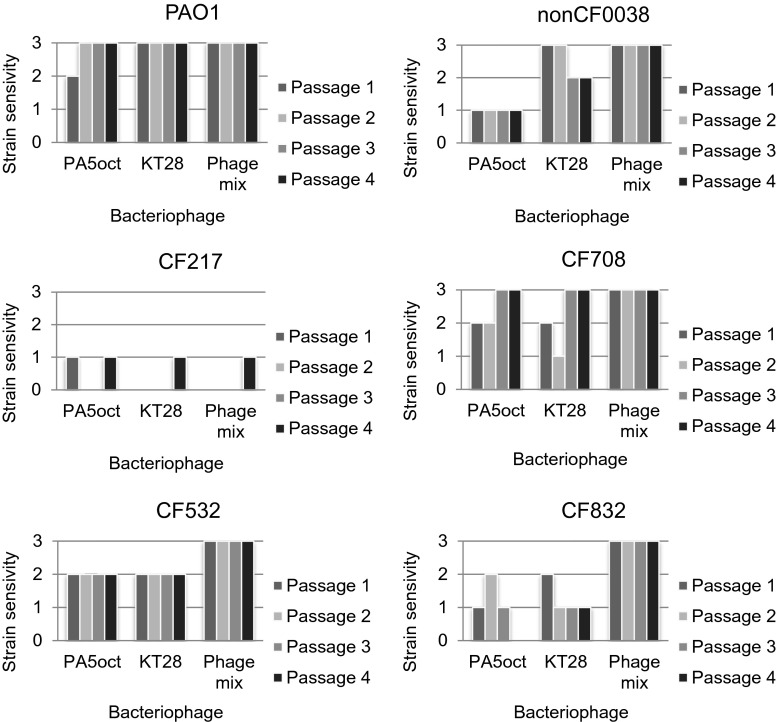
*PA* strains sensitivity variation to selected bacteriophages during four passages. The strain sensitivity denotes confluent clear lysis (*3*), confluent opaque lysis (*2*), single resistant colonies on plaque (*1*), and resistance to phage (*0*)

To explore possible variation in phage susceptibility within a given strain, six *PA* isolates were chosen: PAO1 as a reference strain; non-CF0038, a clinical strain isolated from a wound infection; CF217 (unique cluster, small colony, early infection); CF708 (cluster 1, small colony, slow growth, late infection), and two sequential isolates CF532 nonmucoid and CF832 mucoid from one CF patient (Table 1). The variability of phage lytic activity examined in four bacterial culture passages is illustrated in Fig. 4. Reference PAO1, the non-CF strain, and the paired isolate CF532 exhibited almost homogeneous sensitivity to phages whereas CF217, CF708, and CF832 isolates showed clonal diversity during four passages of the phage treatment assay. The PAO1 and CF532 were fully sensitive to both phages, giving confluent clear lysis in almost all passages. The non-CF isolate (0038) was also susceptible to phage lysis, but in the case of PA5oct application, single resistant colonies were detected in the plaque zone. The CF708 and CF832 were generally susceptible to the chosen phages but exhibited various profiles during four passages of treatment, which suggested strong clonal diversity. The CF217 isolate was almost totally resistant to the applied phages, although phage PA5oct and KT28 gave a slightly inhibitory effect on bacterial growth. The application of a phage cocktail composed of both tested viruses showed homogeneous confluent clear lysis in all four passages (PAO1, non-CF0038, CF708, CF532, CF832), suggesting an additive effect of the phage mixture. No activity of the cocktail was observed for *PA* CF217.

**Table 1:** Morphological diversity of selected six *PA* strains tested by the following features: mucoidy, production of specific type IV pili–twitching motility, and biofilm-forming ability, as the elements considered in term of phage susceptibility variation

Strain	Mucoidy	Twitching motility zone diameter (mm)	Biofilm formation OD (590 nm)
PAO1	Negative (stable)	4.6 ± 0.7	1.14 ± 0.23
Non-CF0038	Negative (stable)	30.4 ± 2.5	0.67 ± 0.1
CF217	Negative (stable)	17.2 ± 1.6	1.92 ± 0.56
CF708	Negative (stable)	1.45 ± 0.9	0.15 ± 0.04
CF532	Negative (stable)	3.8 ± 0.6	0.28 ± 0.09
CF832	Positive (stable)	2.9 ± 0.7	0.04 ± 0.02

### *PA* strains diversity

The high morphological diversity among *PA* strains influencing variation in phage susceptibility was further tested by the following features: (i) mucoidy, (ii) production of specific type IV pili–twitching motility, (iii) biofilm-forming ability, and (iv) biochemical composition fluctuation measured by ATR-FTIR spectroscopy. Bacterial morphology diversity tests (i–iii) are presented in Table 1. Five strains were similarly immutable in their lack of mucoidy, with mucoid CF832 being the exception. There was no significant clonal diversity among particular isolates in type IV pilus-dependent motility. The non-CF0038 and CF217 *PA* isolates exhibited a high degree of twitching motility with zone diameters reaching 30.4 ± 2.5 and 17.2 ± 1.6 mm, respectively, within 48 h. The lowest value of motility was presented by *PA* CF708, suggesting lack of type IV pili. The third examined feature was the ability of biofilm formation which ranged from an OD of 0.04 (CF832) up to OD 1.92 (CF217). Testing 24 randomly taken *PA* colonies revealed the strongest clone diversity in biofilm formation of *PA* CF217, which showed a range of OD from 1.36 to 2.48.

**Fig. 5:**
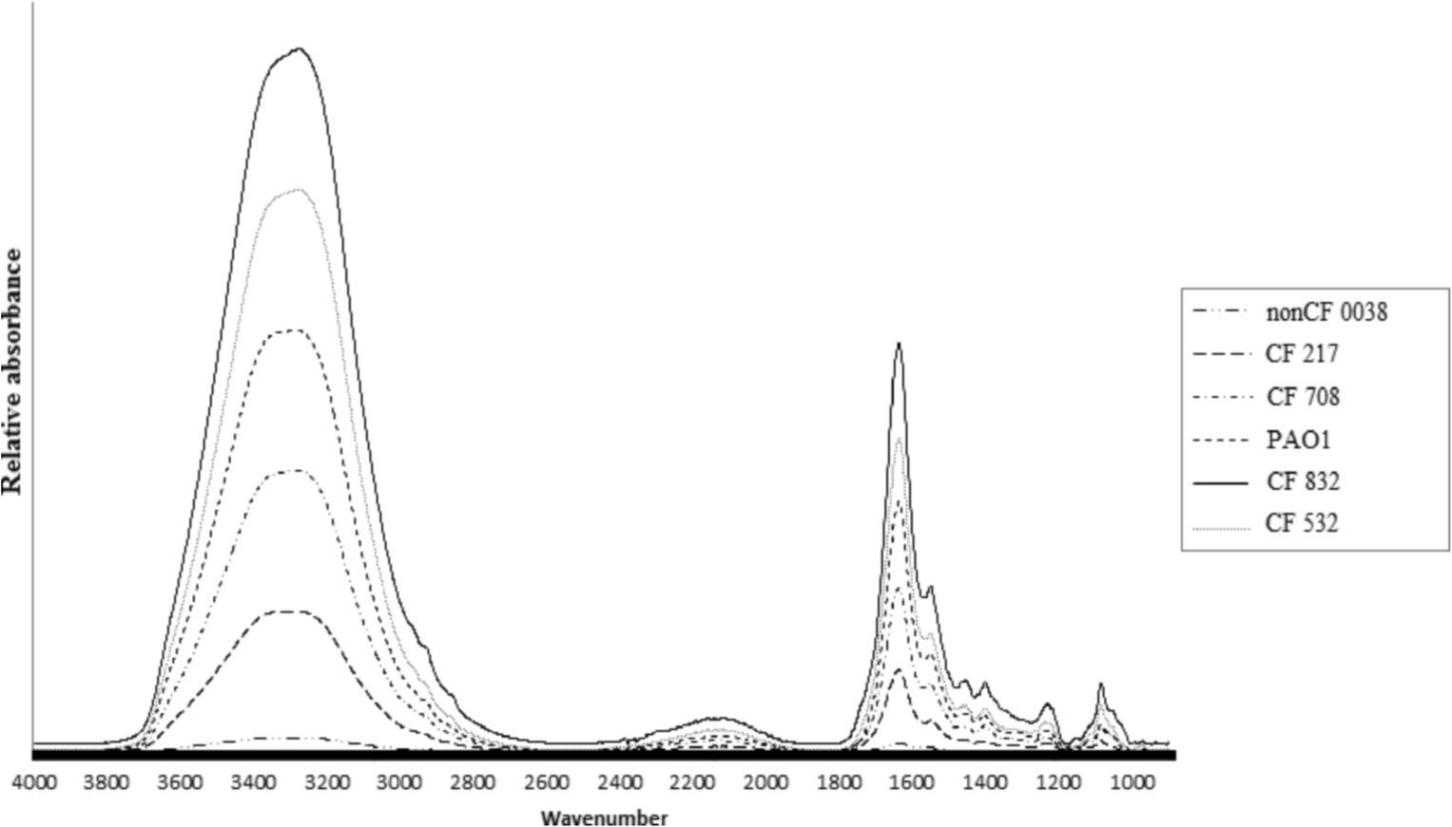
The total chemical profile of six *PA* strains measured by ATR-FTIR spectroscopy over the wavenumber range of 4000 to 900 cm^−1^. Ten single bacterial colonies were tested separately for each strain, and 50 repeats of spectra were collected and averaged to improve the signal to noise ratio

The total chemical profile of bacterial samples was measured by ATR-FTIR spectroscopy over the wavenumber range of 4000 to 900 cm^−1^ (Fig. 5). The vibrations assigned to lipids were around 3000–2800 cm^−1^ (window 1) (Winder and Goodacre 2004); the bands around 1700–1500 cm^−1^ (window 2) derived from proteins and the region from 1450 to 1200 cm^−1^ (window 3) was mainly due to the carboxylic groups of proteins, free amino acids, and polysaccharides wherein wavenumber 1250–1200 cm^−1^ is characteristic for the phosphate stretching mode due to the nucleic acids and phospholipids (San-Blas et al. 2012; Winder and Goodacre 2004). In addition to proteins, lipids, and nucleic acids, carbohydrates are also present, with a typical region of 1200–900 cm^−1^ wavenumber (window 4). For the analyzed strains, the reproducibility of the typing method and the clonal diversity of the bacterial strains were examined based on the mean value of *D* indexes evaluated for all pairs of spectra within a given strain. Reproducibility was measured for the five working ranges (windows 1, 2, 3, 4, and total 4000–900 cm^−1^ wavenumber) (Table 2). The total *D* value among tested strains was relatively low (0.9–4.2), except paired isolates (21.7–24.5). The highest phenotypic clonal variations were noted for the non-CF0038 strain in the lipid window (145.9) and for mucoid strains in the carbohydrates and carboxylic groups spectra (*D* = 70.9 and *D* = 72.8). The most homogeneous culture was the laboratory PAO1 strain, with the *D* value varying from 0.9 to 14.7 in four window spectra. Variability of the *D* value strongly depends on the standardization of sample preparation procedures, spectral data acquisition parameters, medium preparation, growth temperature, incubation time, sample preparation, and spectrum measurement; thus, the appropriate spectral normalization should be included according to Naumann (2000), where mean *D* values up to 10 are considered as standard when analyzing the samples prepared from independently grown cultures and all spectral ranges are analyzed. The obtained results indicated that the crucial differences in colony chemical profile were found in two regions: 3000–2800 cm^−1^ (window 1) and 1200–900 cm^−1^ (window 5) for lipids and carbohydrates, respectively (Table 2). We observed that the variation in phage susceptibility in vitro, measured in four passages treatment, was not related with high *D* values.

**Table 2:** Differentiation index for analyzing biochemical clonal variations within given *P. aeruginosa* strains

Strain	4000–900 cm^−1^ Total spectrum	1200–900 cm^−1^ Window 4 (carbohydrates)	1500–1200 cm^−1^ Window 3 (carboxylic groups)	1700–1500 cm^−1^ Window 2 (proteins)	3000–2800 cm^−1^ Window 1 (lipids)
PAO1	0.9	7.6	6.4	0.3	*14.7*
Non-CF0038	3.0	*17.8*	9.1	1.0	*145.9*
CF217	1.4	9.9	7.7	1.0	*27.8*
CF708	4.2	*32.2*	*16.1*	3.7	*23.5*
CF532	*21.7*	*36.4*	*52.9*	10.1	*111.2*
CF832	*24.5*	*70.9*	*72.8*	9.2	*90.1*

The appropriate spectral normalization according to Naumann (2000) consider *D* values up to 10 as standard. Variability of the *D* value (above 10) are italicized

### In vivo efficacy of phage treatment

To verify the in vitro efficacy of two phages in relation to *PA* strains diversity, the following experiments were done in vivo on the wax moth larvae model. Prior to the appropriate experiments, the virulence of six chosen *PA* strains was determined. *P. aeruginosa* is highly virulent in this model, being their natural pathogen; thus, the lethal doses are very low. The wax moth larvae were injected with serial bacterial inocula (from 10 to 10^8^ CFU) and incubated for 4 days. The caterpillar mortality rate was checked at 8, 24, 48, and 72 h after infection (Table 3). The experiments were controlled by a group of uninfected larvae and a group of larvae that received a control injection of saline where a 100 % survival rate was obtained in both groups. We found that PAO1, non-CF0038, and CF217 *PA* isolates were lethal even at the dose of 10 CFU where over 90 % larvae mortality was noted after 1 day of infection. The CF532, CF832, and CF708 isolates were significantly less virulent in comparison to the most virulent PAO1 (*p* < 0.0001) and even at very high concentrations as 10^5^ CFU dose the delay of killing was observed (100, 90, and 40 % of infected larvae, respectively, after 3 days of infection). These isolates exhibited lower virulence reflected by delay in larvae killing and a slow rate of growth. The lethal doses of 10 CFU for PAO1, non-CF0038 and CF217, and 10^5^ CFU for the remaining strains were chosen for further experiments.

**Table 3:** The virulence of *PA* strains tested in *Galleria* model

	PA01 (10 CFU)	Non-CF0038 (10 CFU)	CF217 (10 CFU)	CF708 (10^5^ CFU)	CF532 (10^5^ CFU)	CF832 (10^5^ CFU)
8 h	80 %	90 %	77 %	100 %	100 %	100 %
24 h	0 %	10 %	6 %	100 %	90 %	100 %
48 h	0 %	0 %	0 %	90 %	40 %	40 %
72 h	0 %	0 %	0 %	60 %	0 %	10 %
96 h	0 %	0 %	0 %	40 %	0 %	0 %

Survival of infected larvae was followed over a period of 4 days. Three independent experiments containing ten larvae per each were pooled. The caterpillar survival rate is presented as a percentage in comparison to 100 % survival rate of uninfected larvae

**Fig. 6:**
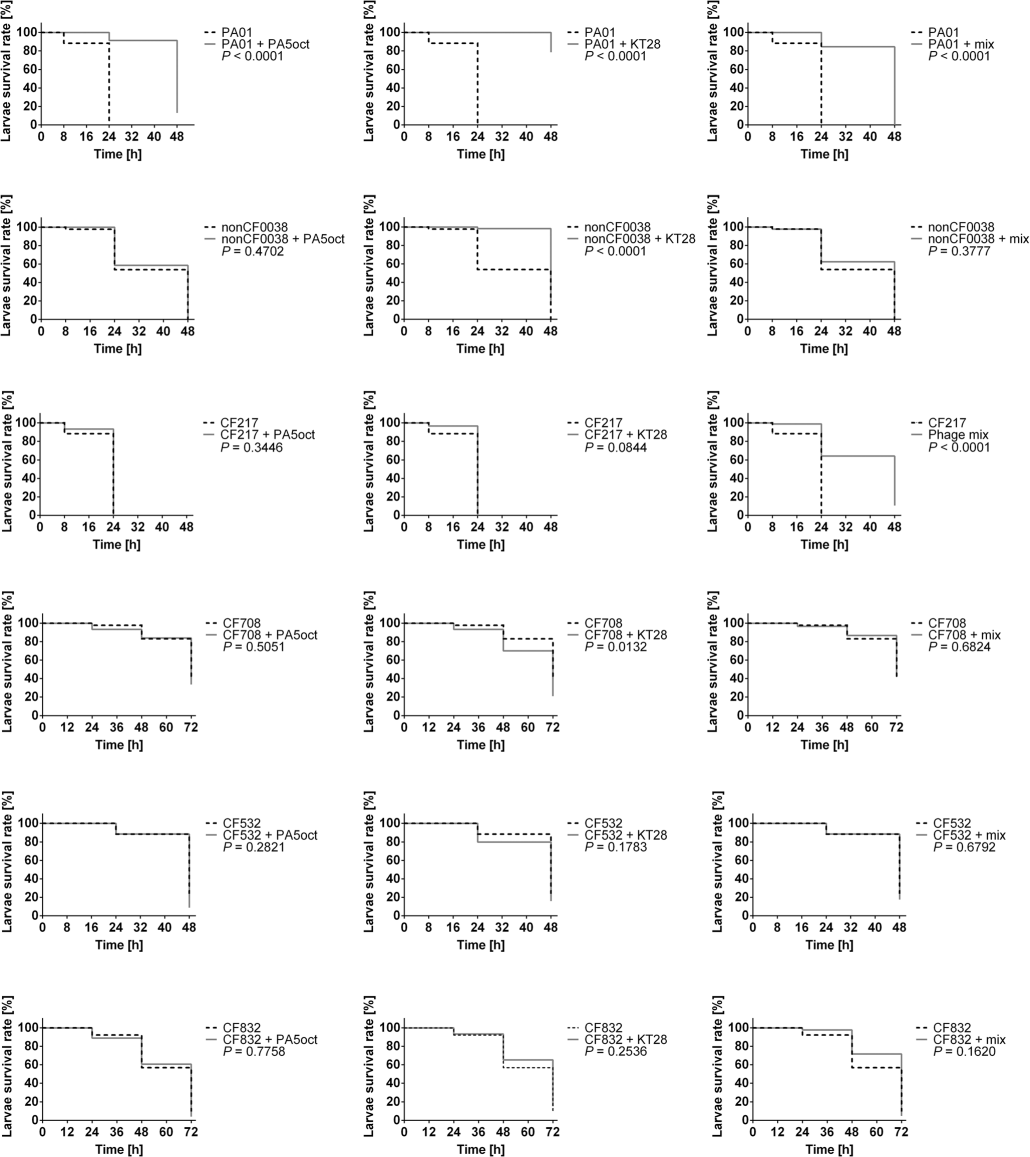
Antibacterial activity of phages (MOI 100) in the treatment of infected *Galleria* larvae by *PA* strains. Survival of infected larvae was followed over a period of 3 days. Three independent experiments containing ten larvae per each were pooled. Statistical analysis was calculated for pairwise comparisons between infected larvae and phage-treated infected larvae using Mantel-Cox test

The phage treatment results were observed at 8, 24, 48, and 72 h after injection (Fig. 6). All experiments were controlled by observation of uninfected larvae, sham-infected larvae, larvae receiving phage lysate only (100 % of larvae survival), and infected but phage untreated larvae. To determine whether the effects of phage therapy were associated with a nonspecific immune activation response, UV-inactivated phages at the same inoculum were used to assess their ability to rescue infected larvae. The efficacy of UV inactivation was assessed if no viable phage was detected by in vitro plating. The inactivated phages were used to treat larvae infected with a lethal dose of *PA* strains, and no differences in larvae mortality were noted in comparison to control infected caterpillars. This confirmed that larval survival was entirely due to phage lytic activity rather than to host immune stimulation.

The phage application showed a significant impact on *Galleria* larval rescue from lethal infection (Fig. 6). Phage KT28 was the most potent whereas PA5oct was the least effective preparation considering both non-CF *Pseudomonas* strains (*p* < 0.0001). The antibacterial activity of the former against non-CF strains showed rescuing around 20 % of larvae even 2 days after lethal dose application. Moreover, KT28 preparation was also more effective against CF708 in comparison to PA5oct (*p* < 0.07). The application of single preparation of KT28 or PA5oct to cure infection of phage resistant strain CF217 was not effective, whereas a mixture of both lysates in the final MOI 100 increased the survival rate of *Galleria* initially infected with CF217 by up to 30 and 20 % at 24 and 48 h, respectively (Fig. 6). However, the protective activity of the phage cocktail against noncystic fibrosis *Pseudomonas* infection in larvae was lower than for single phage application (*p* < 0.05 for mix vs PA5oct against PAO1, *p* < 0.0001 for mix vs KT28 against PAO1 and NS for mix vs PA5oct against non-CF0038, *p* < 0.0001 for mix vs KT28 against non-CF0038).

Unexpected results were obtained for weakly virulent CF isolates (CF708, CF532, CF832). The chosen lethal dose for these isolates was much higher than for previous strains (10^5^ vs 10 CFU); thus, the phage inoculum was also higher to keep MOI value of 100. The application of phage preparation both as single virus or cocktail did not protect larvae from death with one exception (action of KT28 on CF708). The possible explanation of such phenomenon could be the release of toxic compound during massive bacterial cell lysis after phage propagation, which may cause an increase in larvae mortality in comparison to not treated caterpillars.

## Discussion

Recently, phages and phage-encoded proteins have been proposed as natural food preservatives and antimicrobial agents to fight bacterial infections in humans, animals, or crops of agricultural importance (Drulis-Kawa et al. 2012; Glonti et al. 2010). Phage therapy is promising in the eradication of drug-resistant pathogens such as bacteria-colonizing patients suffering from cystic fibrosis (Alemayehu et al. 2012; Carmody et al. 2010; Debarbieux et al. 2010; Essoh et al. 2013; Morello et al. 2011). Recent reports of curative and preventive bacteriophage treatments in a mouse lung infection model, where lytic phages were administered in one single dose, successfully rescued mice from lethal infections caused by *Burkholderia cenocepacia* (Carmody et al. 2010) and *P. aeruginosa* (Morello et al. 2011). Special interest is focused on *P. aeruginosa* infections because of strong clonal diversity of these bacteria which can result in treatment issues. For these reasons, the application of lytic phages was tested in this study on selected *PA* isolates to determine their efficacy in vitro and in vivo in comparison to bacterial diversity of particular biological features such as mucoidy, twitching motility, the intensity of biofilm formation, and biochemical composition. Although strong heterogeneity connected with in vitro phage susceptibility was noted, two myovirus phages with a broad spectrum of activity were selected among 28 environmental phages. The KT28 phage was classified as PB1-like viruses and a novel giant phage PA5oct belonged to the family *Myoviridae* (Drulis-Kawa et al. 2014).

The phenotypical diversity among *PA* strains may influence the variation in phage susceptibility by modifying, masking, or replacing phage receptors required in the first virion-bacterial interactions during viral infection (Abedon 2006; Drulis-Kawa et al. 2012; Guttman et al. 2005; Hanlon 2007; Leclerc et al. 2000; Lenski 1988; Matsuzaki et al. 2005; Young and Wang 2006). Phages can recognize surface components of a bacterial cell including lipopolysaccharide (LPS), peptidoglycan, teichoic acids, outer membrane proteins, oligosaccharides, capsule, and type IV fimbriae for the adhesion process (Abedon 2006; Drulis-Kawa et al. 2012; Guttman et al. 2005; Hanlon 2007; Leclerc et al. 2000; Lenski 1988; Matsuzaki et al. 2005; Young and Wang 2006). The specificity of interaction between phage attachment structures and host cell surface receptors mostly influences the bacterial host range (Drulis-Kawa et al. 2012; Sulakvelidze et al. 2001; Weinbauer 2004). Our previous work done on PAO1 mutants (paper in preparation) allowed the determination of the bacterial receptor recognized by KT28 phage (LPS elements) and PA5oct phage (type IV fimbriae and probably LPS as well). Examining our selected *PA* isolates, we conclude that the PAO1 strain, as the most susceptible to tested phages, was also clonally homogeneous in biochemical analysis done by FTIR and did not produce biofilm intensively. Therefore, this type of host seems to be the best target for effective phage attack. The non-CF *PA* strain (0038) exhibited strong twitching motility and low activity of biofilm formation. Although this strain showed strong clonal variation in lipid composition, the in vitro susceptibility to phages was relatively homogeneous. Lipid composition should have no significant influence on the first step of viral infection, adhesion, because phages usually utilize carbohydrate or protein as receptors (Abedon 2006; Drulis-Kawa et al. 2012; Guttman et al. 2005; Hanlon 2007; Leclerc et al. 2000; Lenski 1988; Matsuzaki et al. 2005; Young and Wang 2006).

Phage lytic activity tested in vitro in the classical agar method was compared with the *Galleria mellonella* larvae in vivo model. This model is a very easy and convenient tool for bacterial virulence determination. Some *P. aeruginosa* strains are highly pathogenic in insects, such as *G. mellonella* (Fancello et al. 2011; Miriagou et al. 2010) and *D. melanogaster* (Barrow and Soothill 1997), when inoculated directly into the hemolymph. In our study, the results obtained with the *G. mellonella* model generally correlated with data obtained from in vitro assays, but only for non-CF strains. The increased larval survival rates in the presence of PB1-like phage, compared to the giant PA5oct, could suggest the importance of phage size and phage generation time rate (KT28 < PA5oct) in the therapeutic results. This needs to be further examined with a range of giant phage isolates. An unexpected feature was seen in the application of phage cocktails. The mixture was less effective in the lysis of phage susceptible *PA* strains PAO1 and non-CF0038 in comparison to single lytic phage administration. A possible explanation for such phenomenon could be the competition between attacking virions for the adhesion receptors on bacterial cells. If the receptors on *PA* surface used by phages are present in a high number and are the same, similar, or located in close vicinity with each other, the effective adhesion of particular virions may be hampered or impaired. This would suggest that phages selected for cocktail preparations should be designed to target different receptors enhancing the host range and allow synergistic activity of phages. Although standardized methods to generate phage cocktails have been proposed (Drulis-Kawa et al. 2012; Merabishvili et al. 2009), no clear official guidelines exist to date (Drulis-Kawa et al. 2012; Pirnay et al. 2011).

The results obtain for CF isolates in this study were more complicated. The CF217 isolate with the growth rate similar to PAO1 strain was almost totally resistant to in vitro administered phages although it produced type IV pili as a potential phage receptor for PA5oct phage. Additionally, the culture exhibited relatively high levels of biofilm production, which might be helpful in the protection against viral invasion. In contrast, in the in vivo study, the cocktail was more efficient in the protection of larvae against *PA* CF217 infection than the application of individual phage, implying an additive effect of mixed preparation in the case of resistant strains (*p* < 0.0001 for mix vs PA5oct against CF217, *p* < 0.0001 for mix vs KT28 against CF217). The strong clonal diversity and loss of virulence of CF-associated *PA* isolates is well known, and our investigation confirms this statement. Slow growth rate, reduced twitching motility, low ability to biofilm formation, and finally strong clonal diversity in the chemical profile of the three tested CF isolates (CF708, CF532, and CF832) significantly influenced the phage efficacy both in vitro and in vivo. The CF708 strain and paired CF532/CF832 isolates were variable in terms of clone carbohydrate and carboxylic group composition, which influenced large differences in phage susceptibility among separate passages (confluent clear and opaque lysis or single resistant colonies on plaque) for CF708 and CF832. Generally, the variations in chemical composition of the culture measured by the FTIR method affected phage efficacy. More complications were noticed in in vivo assay where the application of phage preparation did not cure larvae from infection but even worsened the caterpillar condition (*p* < 0.05 for KT28 against CF708). We suspect that this was due to the massive release of toxic compounds during bacterial cell lysis causing a deterioration of animal health. It is well known that rapid lysis of a big number of cells during phage propagation and the release of LPS from Gram-negative bacteria in a short period of time may cause serious side effects on the host; however, similar effects may occur during bactericidal antibiotic application, as well (Goodridge 2010). The administration of bacteriolytic agents of any kind in the treatment of *Pseudomonas* infection may result in severe consequences as systemic inflammatory response syndrome; thus, the therapy should be carefully selected.

Generally, the treatment success is strongly influenced by heterogeneity of particular pathogens causing chronic infections. The use of lytic phages may lead to selective pressures and drive the expression of undesirable bacterial virulence factors, which must be carefully studied prior to any clinical trials. Overall, the effectiveness of phage cocktails against *PA* isolates is promising alternative but requires additional detailed investigations. The simple animal models such as wax moth larvae model could be a convenient tool to evaluate the effectiveness of particular phage preparations against clinical isolates in vivo. Future work will need to consider the effectiveness of phages in mixed culture models replicating the “airway microbiome” including widely available and well-characterized *PA* strains from diverse sources.

## References

[CR1] Aaron SD, Ferris W, Ramotar K, Vandemheen K, Chan F, Saginur R (2002). Single and combination antibiotic susceptibilities of planktonic, adherent, and biofilm-grown *Pseudomonas aeruginosa* isolates cultured from sputa of adults with cystic fibrosis. J Clin Microbiol.

[CR2] Abedon S, Calendar R, Abedon ST (2006). Phage ecology. The bacteriophages.

[CR3] Ackermann HW, Clokie MRJ, Kropinski AM (2009). Basic phage electron microscopy. Bacteriophages. Methods and protocols. Volume 1: isolation, characterization, and interactions.

[CR4] Adams MH, Adams MH (1959). Bacteriophages. Bacteriophages.

[CR5] Alemayehu D, Casey PG, Mcauliffe O (2012). Bacteriophages ϕMR299-2 and ϕNH-4 can eliminate *Pseudomonas aeruginosa* in the murine lung and on cystic fibrosis lung airway cell. MBio.

[CR6] Alvarez-Ordóñez A, Mouwen DJM, López M, Prieto M (2011). Fourier transform infrared spectroscopy as a tool to characterize molecular composition and stress response in foodborne pathogenic bacteria. J Microbiol Methods.

[CR7] Barrow PA, Soothill JS (1997). Bacteriophage therapy and prophylaxis: rediscovery and renewed assessment of potential. Trends Microbiol.

[CR8] Bradbury R, Champion A, Reid DW (2008). Poor clinical outcomes associated with a multi-drug resistant clonal strain of *Pseudomonas aeruginosa* in the Tasmanian cystic fibrosis population. Respirology.

[CR9] Bragonzi A, Paroni M, Nonis A, Cramer N, Montanari S, Rejman J, Di Serio C, Döring G, Tümmler B (2009). *Pseudomonas aeruginosa* microevolution during cystic fibrosis lung infection establishes clones with adapted virulence. Am J Respir Crit Care Med.

[CR10] Callaghan M, McClean S (2012). Bacterial host interactions in cystic fibrosis. Curr Opin Microbiol.

[CR11] Carmody LA, Gill JJ, Summer EJ, Sajjan US, Gonzales CF, Young RF, LiPuma JJ (2010). Efficacy of bacteriophage therapy in a model of *Burkholderia cenocepacia* pulmonary infection. J Infect Dis.

[CR12] Ceyssens P-J, Miroshnikov K, Mattheus W, Krylov V, Robben J, Noben J-P, Vanderschraeghe S, Sykilinda N, Kropinski AM, Volckaert G, Mesyanzhinov V, Lavigne R (2009). Comparative analysis of the widespread and conserved PB1-like viruses infecting *Pseudomonas aeruginosa*. Environ Microbiol.

[CR13] Cheng K, Smyth RL, Govan JR, Doherty C, Winstanley C, Denning N, Heaf DP, van Saene H, Hart CA (1996). Spread of β-lactam-resistant *Pseudomonas aeruginosa* in a cystic fibrosis clinic. Lancet.

[CR14] Costello A, Herbert G, Fabunmi L, Schaffer K, Kavanagh K, Caraher EM, Callaghan M, McClean S (2011). Virulence of an emerging respiratory pathogen, genus *Pandoraea*, in vivo and its interactions with lung epithelial cells. J Med Microbiol.

[CR15] Cramer N, Klockgether J, Wrasman K, Schmidt M, Davenport CF, Tümmler B (2011). Microevolution of the major common *Pseudomonas aeruginosa* clones C and PA14 in cystic fibrosis lungs. Environ Microbiol.

[CR16] Davies JC (2002). *Pseudomonas aeruginosa* in cystic fibrosis: pathogenesis and persistence. Paediatr Respir Rev.

[CR17] Debarbieux L, Leduc D, Maura D, Morello E, Criscuolo A, Grossi O, Balloy V, Touqui L (2010). Bacteriophages can treat and prevent *Pseudomonas aeruginosa* lung infections. J Infect Dis.

[CR18] Drulis-Kawa Z, Majkowska-Skrobek G, Maciejewska B, Delattre A-S, Lavigne R (2012). Learning from bacteriophages—advantages and limitations of phage and phage-encoded protein applications. Curr Protein Pept Sci.

[CR19] Drulis-Kawa Z, Olszak T, Danis K, Majkowska-Skrobek G, Ackermann H-W (2014). A giant *Pseudomonas* phage from Poland. Arch Virol.

[CR20] Essoh C, Blouin Y, Loukou G, Cablanmian A, Lathro S, Kutter E, Thien HV, Vergnaud G, Pourcel C (2013). The susceptibility of *Pseudomonas aeruginosa* strains from cystic fibrosis patients to bacteriophages. PLoS One.

[CR21] Fancello L, Desnues C, Raoult D, Rolain JM (2011). Bacteriophages and diffusion of genes encoding antimicrobial resistance in cystic fibrosis sputum microbiota. J Antimicrob Chemother.

[CR22] Fothergill JL, Walshaw MJ, Winstanley C (2012). Transmissible strains of *Pseudomonas aeruginosa* in cystic fibrosis lung infections. Eur Respir J.

[CR23] Glonti T, Chanishvili N, Taylor PW (2010). Bacteriophage-derived enzyme that depolymerizes the alginic acid capsule associated with cystic fibrosis isolates of *Pseudomonas aeruginosa*. J Appl Microbiol.

[CR24] Goodridge LD (2010). Designing phage therapeutics. Curr Pharm Biotechnol.

[CR25] Guttman B, Raya R, Kutter E, Kutter E, Sulakvelidze A (2005). Basic phage biology. Bacteriophages biology and application.

[CR26] Hanlon GW (2007). Bacteriophages: an appraisal of their role in the treatment of bacterial infections. Int J Antimicrob Agents.

[CR27] Kropinski AM, Prangishvili D, Lavigne R (2009). Position paper: the creation of a rational scheme for the nomenclature of viruses of bacteria and archaea. Environ Microbiol.

[CR28] Kutter E (2009). Phage host range and efficiency of plating. Methods Mol Biol.

[CR29] Leclerc H, Edberg S, Pierzo V, Delattre JM (2000). Bacteriophages as indicators of enteric viruses and public health risk in groundwaters. J Appl Microbiol.

[CR30] Lenski RE (1988). Dynamics of interactions between bacteria and virulent bacteriophage. Adv Microb Ecol.

[CR31] Maiden MC, Bygraves JA, Feil E, Morelli G, Russell JE, Urwin R, Zhang Q, Zhou J, Zurth K, Caugant DA, Feavers IM, Achtman M, Spratt BG (1998). Multilocus sequence typing: a portable approach to the identification of clones within populations of pathogenic microorganisms. Proc Natl Acad Sci U S A.

[CR32] Matsuzaki S, Rashel M, Uchiyama J, Sakurai S, Ujihara T, Kuroda M, Ikeuchi M, Tani T, Fujieda M, Wakiguchi H, Imai S (2005). Bacteriophage therapy: a revitalized therapy against bacterial infectious diseases. J Infect Chemother.

[CR33] Merabishvili M, Pirnay J-P, Verbeken G, Chanishvili N, Tediashvili M, Lashkhi N, Glonti T, Krylov V, Mast J, Van Parys L, Lavigne R, Volckaert G, Mattheus W, Verween G, De Corte P, Rose T, Jennes S, Zizi M, De Vos D, Vaneechoutte M (2009). Quality-controlled small-scale production of a well-defined bacteriophage cocktail for use in human clinical trials. PLoS One.

[CR34] Miriagou V, Cornaglia G, Edelstein M, Galani I, Giske CG, Gniadkowski M, Malamou-Lada E, Martinez-Martinez L, Navarro F, Nordmann P, Peixe L, Pournaras S, Rossolini GM, Tsakris A, Vatopoulos A, Cantón R (2010). Acquired carbapenemases in Gram-negative bacterial pathogens: detection and surveillance issues. Clin Microbiol Infect.

[CR35] Morello E, Saussereau E, Maura D, Huerre M, Touqui L, Debarbieux L (2011). Pulmonary bacteriophage therapy on *Pseudomonas aeruginosa* cystic fibrosis strains: first steps towards treatment and prevention. PLoS One.

[CR36] Mouwen DJM, Weijtens MJBM, Capita R, Alonso-Calleja C, Prieto M (2005). Discrimination of enterobacterial repetitive intergenic consensus PCR types of *Campylobacter coli* and *Campylobacter jejuni* by Fourier transform infrared spectroscopy. Appl Environ Microbiol.

[CR37] Naumann D, Meyers RA (2000). Infrared spectroscopy in microbiology. Encyclopedia of analytical chemistry.

[CR38] O’Toole GA, Kolter R (1998). Flagellar and twitching motility are necessary for *Pseudomonas aeruginosa* biofilm development. Mol Microbiol.

[CR39] Parkins MD, Rendall JC, Elborn JS (2012). Incidence and risk factors for pulmonary exacerbation treatment failures in patients with cystic fibrosis chronically infected with *Pseudomonas aeruginosa*. Chest.

[CR40] Pirnay J-P, De Vos D, Verbeken G, Merabishvili M, Chanishvili N, Vaneechoutte M, Zizi M, Laire G, Lavigne R, Huys I, Van den Mooter G, Buckling A, Debarbieux L, Pouillot F, Azeredo J, Kutter E, Dublanchet A, Górski A, Adamia R (2011). The phage therapy paradigm: prêt-à-porter or sur-mesure?. Pharm Res.

[CR41] R Core Team (2012). R: a language and environment for statistical computing.

[CR42] San-Blas E, Cubillán N, Guerra M, Portillo E, Esteves I (2012). Characterization of *Xenorhabdus* and *Photorhabdus* bacteria by Fourier transform mid-infrared spectroscopy with attenuated total reflection (FT-IR/ATR). Spectrochim Acta A Mol Biomol Spectrosc.

[CR43] Semmler AB, Whitchurch CB, Mattick JS (1999). A re-examination of twitching motility in *Pseudomonas aeruginosa*. Microbiology.

[CR44] Silbert S, Barth AL, Sader HS (2001). Heterogeneity of *Pseudomonas aeruginosa* in Brazilian cystic fibrosis patients. J Clin Microbiol.

[CR45] Sulakvelidze A, Alavidze Z, Morris JG (2001). Bacteriophage therapy minireview. Antimicrob Agents Chemother.

[CR46] Waszczuk K, Gula G, Swiatkowski M, Olszewski J, Herwich W, Drulis-Kawa Z, Gutowicz J, Gotszalk T (2012). Evaluation of *Pseudomonas aeruginosa* biofilm formation using piezoelectric tuning fork mass sensors. Sensors Actuators B Chem.

[CR47] Weinbauer MG (2004). Ecology of prokaryotic viruses. FEMS Microbiol Rev.

[CR48] Winder CL, Goodacre R (2004). Comparison of diffuse-reflectance absorbance and attenuated total reflectance FT-IR for the discrimination of bacteria. Analyst.

[CR49] Young R, Wang IN, Calendar R, Abedon ST (2006). Phage Lysis. The bacteriophages.

